# Influence of Active Surveillance on Gleason Score Upgrade and Prognosis in Low- and Favorable Intermediate-Risk Prostate Cancer

**DOI:** 10.3390/curroncol29100630

**Published:** 2022-10-21

**Authors:** Xuanhan Hu, Jia Miao, Jiaqing Huang, Lin Qian, Dahong Zhang, Haibin Wei

**Affiliations:** 1 The Second Clinical Medical College, Zhejiang Chinese Medical University, Hangzhou 310053, China; 2 Department of Urology, Taizhou First People’s Hospital, Taizhou 318020, China; 3 Urology & Nephrology Center, Department of Urology, Zhejiang Provincial People’s Hospital (Affiliated People’s Hospital, Hangzhou Medical College), No. 158, Shangtang Road, Xiacheng District, Hangzhou 310014, China

**Keywords:** active surveillance, Gleason score upgrade, favorable intermediate-risk prostate cancer, low-risk prostate cancer, risk stratification

## Abstract

Few studies have focused on the link between active surveillance (AS) and Gleason score upgrade (GSU) and its impact on the prognosis of patients with prostate cancer (PCa). This study aimed to analyze the effect of AS duration on GSU and prognostic value based on risk stratification. All eligible patients were risk-stratified according to AUA guidelines into low-risk (LR), favorable intermediate-risk (FIR), and unfavorable intermediate-risk (UIR) PCa. Within the Surveillance, Epidemiology, and End Results Program (SEER) database, 28,368 LR, 27,243 FIR, and 12,210 UIR PCa patients were included. The relationship between AS duration and GSU was identified with univariate and multivariate logistic regression. Discrimination according to risk stratification of AS duration and GSU was tested by Kaplan–Meier analysis and competing risk regression models. The proportion of patients who chose AS was the highest among LR PCa (3434, 12.1%), while the proportion in UIR PCa was the lowest (887, 7.3%). The AS duration was only associated with GSU in LR PCa, with a high Gleason score (GS) at diagnosis being a strong predictor of GSU for FIR and UIR PCa. Kaplan–Meier analysis indicated that long-term surveillance only made a significant difference in prognosis in UIR PCa. The competing risk analysis indicated that once GS was upgraded to 8 or above, the prognosis in each group was significantly worse. AS is recommended for LR and FIR PCa until GS is upgraded to 8, but AS may not be suitable for some UIR PCa patients.

## 1. Introduction

Prostate cancer (PCa) is the most commonly occurring male cancer in the U.S., accounting for 26%, according to Cancer Statistics 2021 [1]. Currently, active surveillance (AS) has become the primary management modality for clinically localized PCa, especially for low-risk (LR) PCa, as it helps to preserve the patient’s urinary and sexual function to avoid excessive medical treatment [2]. In recent years, the use of AS in LR PCa has increased significantly worldwide, and its effectiveness has been further confirmed [3]. Depending on the National Comprehensive Cancer Network (NCCN) stratification, AS is also recommended for the management of favorable intermediate-risk (FIR) PCa [4].

In previous studies, cancer-specific mortality (CSM) was not worse and remained low (0–1%) in PCa patients with AS compared with surgery or radiation [5]. However, once some patients stopped AS and underwent surgery, Gleason score upgrade (GSU) occasionally occurred during subsequent radical surgery [6]. Corcoran et al. proposed that PCa with GSU was more aggressive and had a higher possibility of biochemical recurrence after radical surgery [7]. A clinical trial proposed that for patients who chose AS, tumor extension beyond the capsule and high post-operative GS often represented a poor prognosis and required endocrine therapy [8]. This suggested that when the tumor progresses, the timing of stopping surveillance is particularly important, which requires further evaluation and trade-offs when needed.

A series of studies have reported the predictors of GSU. German et al. suggested that small prostate size and aging were associated with GSU [9], and Zhang et al. proposed that higher clinical T stage increased the risk of GSU in the cohort with GS = 6 [10]. However, most of these studies have small samples and do not address factors such as AS duration and stratification of risks, which are critical for patients choosing AS. By stratifying the AS duration and the risk of PCa we can develop individualized AS programs to prevent patients from pathological upgrading or poor prognosis due to long-term monitoring

This study analyzed the effect of AS duration on GSU and prognostic value for patients with LR, favorable intermediate-risk (FIR), and unfavorable intermediate-risk (UIR) PCa through the Surveillance, Epidemiology, and End Results Program (SEER) database. A detailed understanding of AS and its impact on prognostic outcomes will help clinicians make better interventional treatment choices for such patients during initial diagnosis and AS.

## 2. Method

### 2.1. Study Population

The SEER database is the largest publicly available oncology database in the United States, covering 34.6% of the U.S. population. The dataset used in this study was extracted from the SEER Research Plus Data, 17 Registries, November 2021 Sub (2000–2019). Since the GS of PCa is included only after 2010, we concentrated on the data between 2010 and 2017. The inclusion criteria for the data are as follows: (1) The primary site was the prostate. (2) The initial primary tumor was confirmed to be prostate carcinoma. (3) Only 3+3, 3+4, and 4+3 GSs obtained from prostate biopsy were included in this study. (4) Tumors were microscopically confirmed. Patients were excluded if they did not undergo a radical prostatectomy, if they had a GS downgrade, or if GS survival time and vital status were unknown.

All eligible patients were risk-stratified according to American Urological Association (AUA) guidelines [11]. LR PCa was defined as PSA < 10 ng/mL, grade group 1, and clinical stage T1-T2a. FIR PCa was described as grade group 1 (with PSA 10–20 ng/mL) or grade group 2 (PSA <10 ng/mL). UIR PCa was defined as grade group 2 (with either PSA 10–20 ng/mL or clinical stage T2b-c) or grade group 3 (PSA < 20 ng/mL). GS was assigned according to the International Society of Urological Pathology (ISUP) grade group designations following current practices [12].

### 2.2. Description of Covariates

Variables analyzed in this study included age at diagnosis, positive cores/biopsy, number of cores per biopsy, percentage of positive biopsy cores, PSA at diagnosis, pathological T stage (AJCC, 7th edition, 2010), pathological N stage (AJCC, 7th edition, 2010), race, AS duration, GS from needle biopsy, GS from the radical surgical specimen, GS upgrade, radiotherapy, lymphodissection, and marriage. Based on the difference in AS duration, we defined AS duration as follows: ”<1 month”, “>1 and ≤5 months”, “>6 and ≤11 months”, “>1 year and ≤2 years”, “>2 years”. For GS from needle biopsy, we divided GS as “3+3”, “3+4”, and “4+3”. PSA at diagnosis was categorized as “<4 ng/mL”, “≥4 and <10 ng/mL”, and “≥10 ng/mL and <20 ng/mL”. The race was classified as “White”, “African American”, “Asian”, and “unknown”.

### 2.3. Statistical Analysis

All data in this study were extracted from the SEER database through SEER*Stat (8.4.0). Descriptive statistical methods were utilized to summarize the demographic characteristics of patients with LR, FIR, and UIR PCa. The Kaplan–Meier method and the log-rank test were used to evaluate the survival function based on the AS duration and GS from the needle biopsy. The predictors related to GSU were screened using a univariate and multivariate logistic regression risk model. Predictors of CSM for LR, FIR, and UIR PCa patients were identified using a competing risk model. *p* values < 0.05 were considered significant. Statistical analyses were performed using IBM SPSS software (v25.0) and the R package (v4.1.1).

## 3. Results

### 3.1. Demographic Features

A total of 67,821 PCa patients were registered from the SEER database from 2010 to 2017. In this series, 57,595 (85.0%) patients received AS, amongst which the largest number were monitored for less than six months (51,015, 88.6%), while only 465 (0.8%) patients were monitored for more than two years.

Table 1 describes the demographic characteristics of patients with PCa according to the risk stratification. LR PCa was more likely to develop GSU, with GS upgraded to “3+4” being the most common. Among the three groups, UIR PCa patients had GS upgraded to 8 or higher. In addition, LR PCa patients preferred long-term AS compared with FIR and UIR PCa. Among all patients, a total of 2820 patients died, of which 407 were cancer-specific. Among those who chose AS, 2327 died, of which 339 were cancer-specific.

### 3.2. Predictors of GSU

Univariate and multivariate logistic regression analyses were performed to analyze the predictors of the risk of PCa (Table 2). We observed that factors such as AS duration, positive cores, and marriage were strongly associated with GSU only in LR PCa, but not in FIR and UIR PCa. The GS obtained by needle biopsy was negatively correlated with GSU in FIR and UIR PCa. In addition, aging and higher PSA at diagnosis were associated with GSU in three groups.

### 3.3. Associations between AS, GSU, and Survival Outcomes

**Figure 1:**
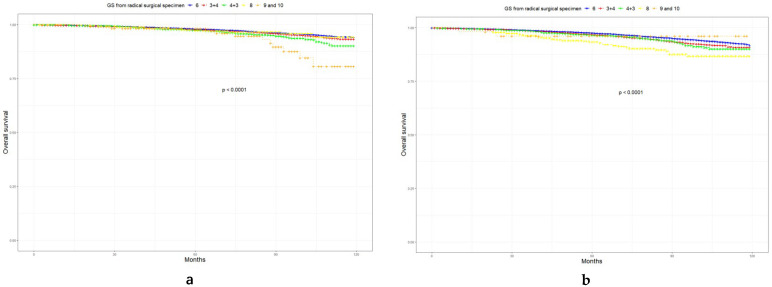

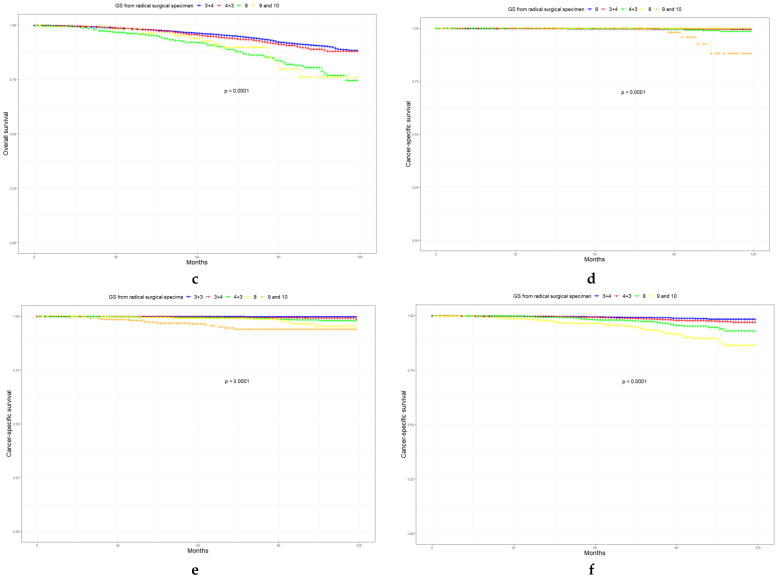
Kaplan–Meier curves of OS and CSS according to GS from radical surgical specimen. (**a**) OS in LR PCa; (**b**) OS in FIR PCa; (**c**) OS in UIR PCa; (**d**) CSS in LR PCa; (**e**) CSS in FIR PCa; (**f**) CSS in UIR PCa. OS, overall survival; CSS, cancer-specific survival; LR PCa, low-risk prostate cancer; FIR PCa, favorable intermediate-risk prostate cancer; UIR PCa, unfavorable intermediate-risk prostate cancer.

**Figure 2:**
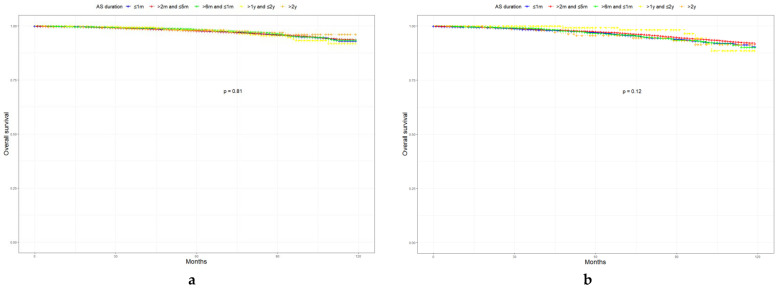

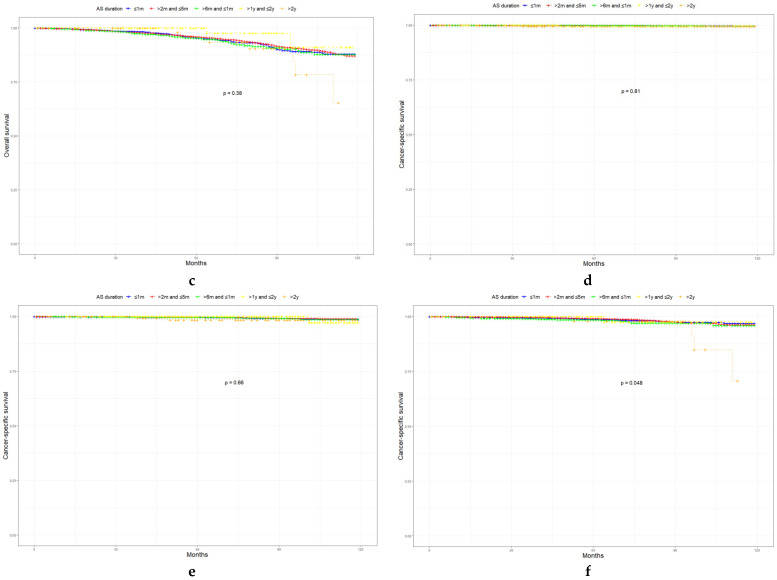
Kaplan–Meier curves of OS and CSS according to AS duration. (**a**) OS in LR PCa; (**b**) OS in FIR PCa; (**c**) OS in UIR PCa; (**d**) CSS in LR PCa; (**e**) CSS in FIR PCa; (**f**) CSS in UIR PCa. OS, overall survival; CSS, cancer-specific survival; LR PCa, low-risk prostate cancer; FIR PCa, favorable intermediate-risk prostate cancer; UIR PCa, unfavorable intermediate-risk prostate cancer.

In this study, 4.1% of patients died, while cancer-specific death accounted for 0.6%. Although LR PCa is most likely to develop GSU, only patients with GS upgraded to 9 or above had significant differences in cancer-specific survival (CSS, HR = 2.49, *p* < 0.01, Figure 1). In FIR and UIR PCa, we found that CSS decreased significantly once GS progressed to 8 (HR = 2.52, *p* < 0.01; HR = 1.92, *p* < 0.01). Subsequently, we analyzed the relationship between AS duration and patient survival (Figure 2). In LR PCa, there was no statistically significant association between AS duration and prognosis (*p* = 0.81). In medium-risk PCa, short-term AS did not change the prognosis of patients. Furthermore, more than two years of monitoring (HR = 3.49, *p* = 0.02) might reduce CSS in UIR PCa.

### 3.4. Prognosis of LR, FIR, and UIR PCa

CSM was measured using a competing risk regression analysis (Table 3). There were 82 (0.2%), 147 (0.5%), and 178 (1.4%) cancer-specific deaths in LR PCa, FIR PCa, and UIR PCa, respectively. In FIR PCa, GS upgraded to 8 and above from the radical surgical specimen was positively correlated with CSM, while in UIR PCa, GS upgrade to 4+3 led to a poor prognosis. Consistent with the survival analysis, AS duration only affected CSM in UIR PCa for more than two years. The pN stage correlated with poorer CSM in FIR and UIR PCa, while radiation therapy was positively associated with CSM in FIR PCa.

## 4. Discussion

For patients newly diagnosed with PCa, subsequent treatment options and clinical outcomes depend largely on biopsy GS. The accuracy of GS and the pathological upgrade are often related to the prognosis, especially for patients who choose non-surgical treatment or AS. With the continuous promotion of AS programs, AS has become the first choice for LR PCa. Moreover, patients with FIR and UIR PCa are willing to try AS [13]. This trend was also observed in this study, with 16.4% of patients opting for immediate treatment after diagnosis in 2010 and only 11.0% in 2017. Interestingly, there was no statistically significant difference in CSM between patients who experienced AS and those who did not (0.5% vs. 0.6%).

Results of this study showed that aging and higher PSA are risk factors for GSU in all three groups, which is consistent with the previous studies [14,15]. It is worth discussing that in this study, the association between GSU and AS duration was different according to risk stratification. LR PCa was more likely to develop GSU, and with the extension of AS, the possibility of GSU also increased. In contrast, AS duration for FIR and UIR PCa did not affect the occurrence of GSU, although it was not stratified for longer surveillance duration. Jain et al. pointed out that sampling error is the leading cause of the escalation of AS patients, especially in LR PCa [16]. This indicates that the occurrence of GSU is progressing over time. In addition, a research group from Toronto demonstrated that post-operative pathological results of patients undergoing AS were not statistically different from the results of cases that did not undergo AS [17].

The present study indicates that the number of biopsy-positive cores was positively correlated with the occurrence of GSU in LR PCa, which was consistent with the previous research [18]. This phenomenon suggests that although multinucleus biopsies may further reduce the risk of sampling error in prostate biopsies, the heterogeneity and multifocal nature of PCa are the main factors leading to the pathological escalation of LR PCa.

Although AS is widely accepted in LR PCa, it is still controversial for intermediate-risk PCa patients, as they are at higher risk for progression to metastatic disease [19]. Among all patients who died, cardiac disease was the leading cause of death, while cancer-specific death accounted for only 14.4%. We observed that long-term surveillance had no significant effect on patients’ CSM in LR and FIR PCa, while more than two years of monitoring can affect the outcomes of patients in the UIR PCa group. Meissner et al. also proposed that the prognosis of UIR PCa was significantly worse than that of FIR PCa [20]. Built on the above results, it is safer to say that AS is feasible for FIR PCa. However, for UIR PCa, long-term active surveillance may need further evaluation.

Finally, looking at CSM in this patient cohort, we found that the results were consistent with survival analysis. Surveillance for UIR PCa over two years resulted in poor prognosis, with no effect on LR and FIR PCa. However, a study from the Princess Margaret Cancer Centre, Canada, proposed that AS for intermediate-risk PCa over time did not affect the pathologic or oncological outcomes [21]. We attribute this to the selection bias, as in this study, only 68 patients (0.5%) in the UIR PCa group opted to be monitored for more than two years, of which four patients died as a result of cancer-specific factors. According to the above conclusions, we believe that for UIR PCa, the short-term monitoring program can be harmless. Whether long-term monitoring will affect the patient’s prognosis still needs further research.

The authors of previous studies believed that once GSU occurred, it led to post-operative biochemical recurrence and poor prognosis [22]. However, for LR PCa patients, most of them (11,753, 85.8%) were upgraded to 3+4, which did not affect the prognosis. Interestingly, the conclusions of the survival analysis and the competing risk model were different. GSU in competing risk models is more likely to lead to poor outcomes than that in survival analysis, especially for UIR PCa. We speculate that the above differences were due to the inclusion of post-operative indicators in competing risk models, such as pathological stage and post-operative GS, which improved the prognostic assessment. For patients who continue AS, the risk stratification should be strictly followed and observed for tumor progression. The AS regimen can be discontinued once the repeated prostate biopsy indicates GS upgrades to 8 for LR and FIR PCa.

Smith et al. suggested that African American men are at higher risk of tumor progression with a worse prognosis [23]. In the present study, the conclusion was consistent with that of the above-mentioned study, but only in the case of LR PCa. Disease-specific factors have made an impact on outcomes in FIR and UIR PCa. Unmarried patients had higher CSM in LR and FIR PCa. This may be due to a combination of social factors, including cancer screening, mental health, and quality of life [24].

In summary, our study identified the relationship between AS duration and GSU and explored the impact of AS and GSU on CSM. In LR and FIR PCa, when prostate biopsy pathology suggests GS upgrades to 3+4 or 4+3, it can be combined with symptoms and various tests to evaluate whether monitoring should be continued. Once the GS is upgraded to 8 and above, monitoring should be stopped immediately. Long-term AS is inappropriate for UIR PCa, leading to pathological escalation and poor prognosis. Nonetheless, the above conclusions need to be interpreted in a limited context. Since the data in this study were derived from the SEER database, the duration of AS and GS from needle biopsy were only available after 2010. In addition, due to the retrospective nature of this study, data for prostate size, PSA density, and the number of positive MRI-targeted needle biopsies are still missing. Only the GS of the first biopsy was available for patients with long-term monitoring and repeated biopsies. In addition, only 0.8% of the patients chose long-term monitoring in this study, which might have biased the statistical results.

## 5. Conclusions

Stratification of GSU is particularly important for predicting prognosis, particularly for LR and FIR PCa. Once LR and FIR PCa patients’ GS is upgraded to 8 points or above, they have a poorer prognosis, which provides a theoretical basis for the timing of cessation of AS. For UIR PCa, long-term surveillance often results in tumor progression and poor prognosis, and AS may not be appropriate for these patients.

## Figures and Tables

**Table 1:** Patient demographics, stratified by the risk according to AUA guidelines.

Characteristic	Level	Low-Risk Prostate Cancer	Favorable Intermediate-Risk Prostate Cancer	Unfavorable Intermediate-Risk Prostate Cancer	*p*
N		28,368	27,243	12,210	
Age (median (IQR))		60 (55–65)	57 (52–62)	63 (58–68)	<0.001
Year of diagnosis (median (IQR))		2012 (2011–2014)	2013 (2011–2016)	2014 (2012–2016)	<0.001
Positive cores/biopsy (median (IQR))		3 (1–5)	4 (2–6)	5 (3–7)	<0.001
Number of cores per biopsy (median (IQR))		12 (12–12)	12(12–12)	12 (12–12)	<0.001
Percentage of positive biopsy cores (median (IQR))		25.0 (14.3–41.7)	33.3 (21.4–50.0)	41.7 (25.0–62.5)	<0.001
Gleason score from needle biopsy (%)	3+3	28,368 (100.0)	3394 (12.5)	0 (0.0)	<0.001
	3+4	0 (0.0)	23,849 (87.5)	4166 (34.1)	
	4+3	0 (0.0)	0 (0.0)	8044 (65.9)	
Race (%)	White	23,315 (82.2)	22,100 (81.1)	9728 (79.7)	<0.001
	Black	3592 (12.7)	3543 (13.0)	1605 (13.1)	
	Asian	1215 (4.3)	1348 (4.9)	765 (6.3)	
	Unknown	246 (0.9)	252 (0.9)	112 (0.9)	
PSA (%)	<4 ng/mL	5614 (19.8)	2989 (11.0)	695 (5.7)	<0.001
	≥4 and <10 ng/mL	22,754 (80.2)	20,860 (76.6)	5493 (45.0)	
	≥10 ng/mL and <20 ng/mL	0 (0.0)	3394 (12.5)	6022 (49.3)	
pT (%)	T1-T2a	4057 (14.3)	2635 (9.7)	837 (6.9)	<0.001
	T2b-c	21,237 (74.9)	17,549 (64.4)	5541 (45.4)	
	T3-T4	3074 (10.8)	7059 (25.9)	5832 (47.8)	
pN (%)	N0	27,987 (98.7)	26,640 (97.8)	11,371 (93.1)	<0.001
	N1	84 (0.3)	416 (1.5)	792 (6.5)	
	Unknown	297 (1.0)	187 (0.7)	47 (0.4)	
Radiation (%)	No	27,857 (98.2)	26,144 (96.0)	10,979 (89.9)	<0.001
	Yes	511 (1.8)	1099 (4.0)	1231 (10.1)	
Lymphodissection (%)	No	17,820 (62.8)	9060 (33.3)	2314 (19.0)	<0.001
	Yes	10,548 (37.2)	18,183 (66.7)	9896 (81.0)	
Marriage (%)	Unmarried	4543 (16.0)	5048 (18.5)	2526 (20.7)	<0.001
	Married	21,875 (77.1)	20,487 (75.2)	8945 (73.3)	
	Unknown	1950 (6.9)	1708 (6.3)	739 (6.1)	
AS duration (%)	<1 month	4298 (15.2)	4038 (14.8)	1890 (15.5)	<0.001
	≥1 and <5 months	20,636 (72.7)	20,946 (76.9)	9433 (77.3)	
	≥6 and <11 months	2801 (9.9)	1894 (7.0)	739 (6.1)	
	≥1 year and <2 years	390 (1.4)	211 (0.8)	80 (0.7)	
	≥2 years	243 (0.9)	154 (0.6)	68 (0.6)	
Gleason score between the radical surgical specimen (%)	6	14,678 (51.7)	1341 (4.9)	0 (0.0)	<0.001
	3+4	11,753 (41.4)	20,610 (75.7)	2922 (23.9)	
	4+3	1516 (5.3)	4274 (15.7)	7271 (59.5)	
	8	295 (1.0)	605 (2.2)	1062 (8.7)	
	9 and 10	126 (0.4)	413 (1.5)	955 (7.8)	
Gleason score upgrade (%)	0	14,678 (51.7)	20,379 (74.8)	9240 (75.7)	<0.001
	1	11,753 (41.4)	5490 (20.2)	1849 (15.1)	
	2	1516 (5.3)	888 (3.3)	996 (8.2)	
	3	295 (1.0)	434 (1.6)	125 (1.0)	
	4	126 (0.4)	52 (0.2)	0 (0.0)	

**Table 2:** Univariate and multivariate logistic regression specific factors for predicting progress in Gleason score according to risk stratification.

	Univariate				Multivariable			
	Factor	Level	OR (95%CI)	*p*	Factor	Level	OR (95%CI)	*p*
Low-risk prostate cancer	Age		1.03 (1.02–1.04)	<0.001	Age		1.03 (1.02–1.04)	<0.001
	Race	White	reference		Race	White	reference	
		Black	1.09 (1.02–1.17)	0.011		Black	1.14 (1.06–1.22)	<0.001
		Asian	1.33 (1.19–1.50)	<0.001		Asian	1.27 (1.13–1.44)	<0.001
	PSA	<4 ng/mL	reference		PSA	<4 ng/mL		
		≤4 and <10 ng/mL	1.76 (1.66–1.87)	<0.001		≥4 and <10 ng/mL	1.58 (1.48–1.68)	<0.001
	Positive cores/biopsy	≤3	reference		Positive cores/biopsy	≤3	reference	
		>3	1.29 (1.22–1.37)	<0.001		>3	1.32 (1.18–1.47)	<0.001
	Percentage of positive biopsy cores	≤25%	reference		Percentage of positive biopsy cores	≤25%	reference	
		>25%	1.32 (1.25–1.40)	<0.001		>25%	1.41 (1.26–1.57)	<0.001
	AS duration	<1 month	reference		AS duration	<1 month	reference	
		≥1 and <5 months	1.17 (1.09–1.25)	<0.001		≥1 and <5 months	1.18 (1.10–1.27)	<0.001
		≥6 and <11 months	1.35 (1.23–1.49)	<0.001		≥6 and <11 months	1.43 (1.29–1.57)	<0.001
		≥1 year and <2 years	1.80 (1.46–2.22)	<0.001		≥1 year and <2 years	1.91 (1.54–2.36)	<0.001
		≥2 years	1.39 (1.07–1.80)	0.019		≥2 years	1.39 (1.06–1.81)	<0.001
	Marriage	Unmarried	reference					
		Married	0.98 (0.92–1.05)	>0.05				
Favorable intermediate-risk prostate cancer	Age		1.01 (1.01–1.02)	<0.001	Age		1.01 (1.01–1.02)	<0.001
	Race	White	reference					
		Black	1.02 (0.94–1.10)	>0.05				
		Asian	1.12 (0.98–1.26)	0.070				
	PSA	<4 ng/mL	reference		PSA	<4 ng/mL	reference	
		≥4 and <10 ng/mL	1.24 (1.12–1.37)	<0.001		≥4 and <10 ng/mL	1.20 (1.09–1.34)	<0.001
		≥10 ng/mL and <20 ng/ml	7.33 (6.53–8.25)	<0.001		≥10 ng/mL and <20 ng/ml	7.35 (6.53–8.28)	<0.001
	Positive cores/biopsy	<=4	reference		Positive cores/biopsy	≤4	reference	
		>4	0.85 (0.79–0.90)	<0.001		>4		>0.05
	Percentage of positive biopsy cores	≤33.3%	reference		Percentage of positive biopsy cores	≤33.3%	reference	
		>33.3%	0.85 (0.80–0.91)	<0.001		>33.3%	1.13 (1.04–1.24)	<0.001
	Gleason score from needle biopsy	6	reference		Gleason score from needle biopsy	6	reference	
		3+4	0.16 (0.15–0.17)	<0.001		3+4	0.21 (0.13–0.28)	<0.001
	AS duration	<1 month	reference		AS duration	<1 month	reference	
		≥1 and <5 months	1.04 (0.96–1.15)	>0.05		≥1 and <5 months		>0.05
		≥6 and <11 months	1.44 (1.28–1.63)	<0.001		≥6 and <11 months		>0.05
		≥1 year and <2 years	1.37 (1.01–1.85)	0.037		≥1 year and <2 years		>0.05
		≥2 years	1.75 (1.24–2.45)	<0.001		≥2 years		>0.05
	Marriage	Unmarried	reference		Marriage	Unmarried	reference	
		Married	0.91 (0.85–0.98)	0.016		Married		>0.05
Unfavorable intermediate-risk prostate cancer	Age		1.00 (1.00–1.01)	0.032	Age		1.00 (1.00–1.01)	0.012
	Race	White	reference		Race	White	reference	
		Black	0.82 (0.72–0.94)	0.004		Black	0.82 (0.72–0.94)	<0.001
		Asian	1.09 (0.92–1.29)	>0.05		Asian		>0.05
	PSA	<4 ng/mL	reference		PSA	<4 ng/mL	reference	
		≥4 and <10 ng/mL	0.81 (0.67–0.97)	0.023		≥4 and <10 ng/mL	0.80 (0.70–0.91)	0.022
		≥10 ng/mL and <20 ng/ml	1.26 (1.05–1.52)	0.011		≥10 ng/mL and <20 ng/ml	1.28 (1.06–1.54)	0.008
	Positive cores/biopsy	<=5	reference		Positive cores/biopsy	≤5	reference	
		>5	1.11 (1.01–1.22)	0.018		>5		>0.05
	Percentage of positive biopsy cores	≤41.7%	reference		Percentage of positive biopsy cores	≤41.7%	reference	
		>41.7%	1.15 (1.04–1.26)	0.003		>41.7%		>0.05
	Gleason score from needle biopsy	3+4	reference		Gleason score from needle biopsy	3+4	reference	
		4+3	0.64 (0.58–0.70)			4+3	0.75 (0.66–0.85)	<0.001
	AS duration	<1 month	reference					
		≥1 and <5 months		>0.05				
		≥6 and <11 months		>0.05				
		≥1 year and <2 years		>0.05				
		≥2 years		>0.05				
	Marriage	Unmarried	reference					
		Married		>0.05				

Abbreviations: OR: odds ratio, CI: confidence interval.

**Table 3:** Competing risk regression analysis predicting cancer-specific mortality in patients with prostate cancer according to risk stratification.

	Characteristic	HR (95%CI)	*p*
Low-risk prostate cancer	Age		>0.05
	Time from diagnosis to treatment		
	≤1 month	reference	
	>1 and ≤5 months		>0.05
	>6 and ≤11 months		>0.05
	>1 year and ≤2 years		>0.05
	>2 years		>0.05
	Race		
	White	reference	
	Black	2.27 (1.37–3.78)	0.001
	Asian		>0.05
	pT		
	T1-T2a	reference	
	T2b-c		>0.05
	T3-T4		>0.05
	pN		
	N0	reference	
	N1		>0.05
	GS from the radical surgical specimen		
	6	reference	
	3+4		>0.05
	4+3		>0.05
	8	7.10 (4.61–11.0)	<0.001
	9 and 10	7.55 (2.29–24.8)	<0.001
	Lymphodissection		
	No	reference	
	Yes	1.56 (1.00–2.42)	0.048
	Radiotherapy		
	No	reference	
	Yes		>0.05
	Marriage		
	Unmarried	reference	
	Married	0.52 (0.32–0.89)	0.016
	Unknown		>0.05
Favorable intermediate-risk prostate cancer	Age	1.06 (1.03–1.08)	<0.001
	Time from diagnosis to treatment		
	≤1 month	reference	
	>1 and ≤5 months		>0.05
	>6 and ≤11 months		>0.05
	>1 year and ≤2 years		>0.05
	>2 years		>0.05
	Race		
	White	reference	
	Black		>0.05
	Asian		>0.05
	pT		
	T1-T2a	reference	
	T2b-c		>0.05
	T3-T4		>0.05
	pN		
	N0	reference	
	N1	2.51 (1.22–5.16)	0.012
	GS from the radical surgical specimen		
	6	reference	
	3+4		>0.05
	4+3		>0.05
	8	2.52 (1.84–3.42)	0.016
	9 and 10	11.3 (3.82–23.4)	<0.001
	Lymphodissection		
	No	reference	
	Yes		>0.05
	Radiotherapy		
	No	reference	
	Yes	1.90 (1.10–3.29)	0.022
	Marriage		
	Unmarried	reference	
	Married	0.45 (0.29–0.70)	<0.001
	Unknown		>0.05
Unfavorable intermediate-risk prostate cancer	Age		>0.05
	Time from diagnosis to treatment		
	≤1 month	reference	
	>1 and ≤5 months		>0.05
	>6 and ≤11 months		>0.05
	>1 year and ≤2 years		>0.05
	>2 years	4.54 (1.59–9.55)	0.003
	Race		
	White	reference	
	Black		>0.05
	Asian		>0.05
	pT		
	T1-T2a	reference	
	T2b-c		>0.05
	T3-T4	5.95 (1.89–18.8)	0.004
	pN		
	N0	reference	
	N1	2.51 (1.22–5.16)	<0.001
	GS from the radical surgical specimen		
	3+4	reference	
	4+3	1.76 (1.18–2.63)	0.006
	8	5.53 (3.94–7.78)	<0.001
	9 and 10	3.12 (1.15–8.44)	0.025
	Lymphodissection		
	No	reference	
	Yes		>0.05
	Radiotherapy		
	No	reference	
	Yes		>0.05
	Marriage		
	Unmarried	reference	
	Married		>0.05
	Unknown		>0.05

## Data Availability

The full dataset generated and analyzed during the current study is available from the corresponding author on reasonable request.

## Abbreviations

AS	Active surveillance
PCa	Prostate cancer
GSU	Gleason score upgrade
LR	Low-risk
FIR	Favorable intermediate-risk
UIR	Unfavorable intermediate-risk
NCCN	National Comprehensive Cancer Network
CSM	Cancer-specific mortality
SEER	Surveillance, Epidemiology, and End Results Program
ISUP	International Society of Urological Pathology
AUA	American Urological Association
CSS	Cancer-specific survival
OS	Overall survival
PSA	Prostate-specific antigen
